# Nerve growth factor promotes breast cancer angiogenesis by activating multiple pathways

**DOI:** 10.1186/1476-4598-9-157

**Published:** 2010-06-22

**Authors:** Rodrigue Romon, Eric Adriaenssens, Chann Lagadec, Emmanuelle Germain, Hubert Hondermarck, Xuefen Le Bourhis

**Affiliations:** 1 INSERM U908, F-59650 Villeneuve d'Ascq, France; 2 USTL, F-59650 Villeneuve d'Ascq, France; 3 CNRS UMR 8161, Institut Pasteur de Lille, F-59800 Lille, France; 4 Univ Lille Nord de France, F-59000 Lille, France

## Abstract

**Background:**

Although several anti-angiogenic therapies have been approved in the treatment of cancer, the survival benefits of such therapies are relatively modest. Discovering new molecules and/or better understating signaling pathways of angiogenesis is therefore essential for therapeutic improvements. The objective of the present study was to determine the involvement of nerve growth factor (NGF) in breast cancer angiogenesis and the underlying molecular mechanisms.

**Results:**

We showed that both recombinant NGF and NGF produced by breast cancer cells stimulated angiogenesis in Matrigel plugs in immunodeficient mice. NGF strongly increased invasion, cord formation and the monolayer permeability of endothelial cells. Moreover, NGF-stimulated invasion was under the control of its tyrosine kinase receptor (TrkA) and downstream signaling pathways such as PI3K and ERK, leading to the activation of matrix metalloprotease 2 and nitric oxide synthase. Interestingly, NGF increased the secretion of VEGF in both endothelial and breast cancer cells. Inhibition of VEGF, with a neutralizing antibody, reduced about half of NGF-induced endothelial cell invasion and angiogenesis *in vivo*.

**Conclusions:**

Our findings provided direct evidence that NGF could be an important stimulator for breast cancer angiogenesis. Thus, NGF, as well as the activated signaling pathways, should be regarded as potential new targets for anti-angiogenic therapy against breast cancer.

## Background

It is well established that tumor growth beyond the size of 1-2 mm is dependent upon angiogenesis [1]. This process is regulated by numerous proangiogenic factors which are secreted by tumor or surrounding stromal cells. Among these proangiogenic factors, vascular endothelial growth factor (VEGF) plays a pivotal role in tumor angiogenesis. VEGF promotes angiogenesis *via *its ability to stimulate permeability, growth, migration and invasion of endothelial cells, and to mobilize endothelial precursor cells from bone marrow [2-4]. Inhibition of VEGF reduces angiogenesis and tumor growth *in vivo *[5]. Conversely, VEGF overexpression is associated with increased microvessel density, tumor metastasis, and poor prognosis [6-8]. Among several VEGF isoforms, VEGF-A is the most predominant angiogenic factor, as its level is strongly associated with tumor progression and poor clinical outcome in many types of cancers including breast cancer [9-11].

NGF has been studied most extensively for its role in regulating growth, development, survival and regeneration of the nervous system. NGF exerts its effects through two membrane receptors: the tyrosine kinase receptor TrkA and the neurotrophin receptor p75^NTR^, a common receptor for all neurotrophins and pro-neurotrophins. NGF binding to TrkA induces TrkA receptor dimerisation and autophosphorylation of cytoplasmic tyrosines, leading to the activation of various signaling pathways, including Ras/MAPK, PLCγ, and PI3K/Akt [12,13]. NGF has also been reported to promote angiogenesis and/or induces the expression of proangiogenic molecules in several tissues, such as muscle and cornea [14-16]. On the other hand, NGF has been increasingly described to regulate tumor growth and progression of non-neuronal cancers including medullar thyroid carcinoma [17], lung [18], pancreatic [19], prostatic [20] and breast carcinomas [21-23]. In breast cancers, we have previously shown that NGF and its tyrosine kinase receptor TrkA are overexpressed compared to normal breast tissues [24,25]. Inhibition of NGF with neutralizing antibodies, or small interfering RNA, strongly reduces angiogenesis and tumor development in immunodeficient mice [24]. Conversely, TrkA overexpression in breast cancer cells leads to a constitutive activation of its tyrosine kinase, resulting in increased tumorigenicity as well as enhanced angiogenesis [25]. Similar link between NGF and angiogenesis has also been suggested in ovarian carcinomas [26].

The objective of the present study was to better determine the possible involvement of NGF in breast cancer angiogenesis, as well as the underlying molecular mechanisms. We showed that NGF secreted by breast cancer cells could stimulate tumor angiogenesis *in vivo*. NGF increased growth, migration, invasion, tubular formation and permeability of endothelial cells. We also demonstrated the involvement of multiple pathways such as PI3K-Akt, ERK, MMP2, and NO synthase as well as the role of VEGF in the angiogenic effect of NGF.

## Materials and Methods

### Reagents

Human recombinant NGF and VEGF, neutralizing antibodies against NGF, VEGF and the corresponding isotype control antibodies were purchased from R&D Systems. Growth factor-reduced Matrigel was from BD Biosciences. Cleavage resistant proNGF was from Alomone (Israël).

### Cell Culture

Human umbilical vein endothelial cells (HUVEC) from Lonza were a pool derived from 3 donors. Cells were maintained at 37°C with humidified 95% air/5% CO_2 _in endothelial growth medium (EGM) containing 2% fetal bovine serum (FBS) and other compounds of the EGM singlequots provided with the medium (Lonza). For different experiments, HUVEC were cultured in starved medium composed of endothelial basal medium (EBM) containing 0.5% FBS and GA-1000 (provided in the EGM singlequots). MDA-MB-231 human breast cancer cells from American Type Culture Collection were maintained in Minimal Essential Medium (MEM) supplemented with 20 mM HEPES, 2 g/l sodium bicarbonate, 2 mM L-glutamine, 1% of non-essential amino acids, 10% fetal calf serum (FCS).

### *In vivo *Angiogenesis

Six-week-old female severe combined immunodeficient (SCID) mice were from Institut Pasteur de Lille, France. Mice were maintained in accordance with the Institutional Animal Care and Use Committee procedures and guidelines. Angiogenesis was analyzed by Matrigel plug assay, as described below.

#### Matrigel plug assay

To determine the influence of endogenously produced NGF in breast cancer angiogenesis, cold Matrigel was mixed with MDA-MB-231 breast cancer cells in the presence of isotype control, or anti-NGF neutralizing antibodies (75 μg/ml). To determine the influence of recombinant NGF in angiogenesis, cold Matrigel was mixed with PBS (as control), 3.75 μg/ml NGF, 7.5 μg/ml proNGF, or 0.375 μg/ml VEGF. In some experiments, cold Matrigel was also mixed with 3.75 μg/ml NGF and isotype control or anti-VEGF (37.5 μg/ml) neutralizing antibodies. A total of 500 μl of the mixed Matrigel was subcutaneously injected into SCID mice in the middle lateral dorsal region. Seven days later, the animals were sacrificed and the Matrigel plugs were harvested. Pictures of Matrigel plug were taken with a Sony DSC-W5 numerical camera.

#### Hemoglobin quantification

Hemoglobin quantification was performed as previously described [27]. Briefly, the Matrigel plugs were homogenized in 500 μl water on ice and cleared by centrifugation at 200 g for 6 min at 4°C. The supernatant was collected and used in triplicate to measure hemoglobin content with Drabkin's reagent (Sigma-Aldrich) according to manufacturer instruction. The absorbance was measured at 540 nm.

#### Microvessel density analysis

Matrigel plugs were fixed in 4% paraformaldehyde, embedded in paraffin and sections cut at 3-4 μm intervals. Detection of the specific marker of endothelial cell CD31 by immunohistochemistry was performed with the Renaissance TSA Biotin System kit (PerkinElmer). The antibody used for immunohistochemistry against CD31 was from Novus Biologicals and the corresponding biotinylated anti rat secondary antibody was from BD Pharmingen. The reaction was developed with DAB substrate (Sigma-Aldrich) and sections were counterstained with Mayer's hematoxylin (Sigma-Aldrich). The microvessel density was quantified in 10 vascular hot-spot fields, by determining the area covered by CD31-positive staining, using image analysis, as previously described [28].

### **Endothelial cell behaviour assays in culture**

#### Endothelial cell growth Assay

HUVEC (10^5 ^cells/well) were seeded in six well plates in 2 ml EBM/0.5% FBS and cultured for 24 h. Cells were then treated with 100 ng/ml NGF or 10 ng/ml VEGF for 48 h. They were harvested by trypsinization and counted using a hemocytometer (Coulter Z2, Beckman-Coulter).

#### Endothelial cell migration and invasion

BD Falcon inserts with a polyethylene terephthalate (PET) membrane/8 μm pores (BD Biosciences) were used for migration and invasion assays. The inserts were pre-coated with diluted Matrigel (1:100 for migration and 1:10 for invasion). HUVEC (5.10^4 ^cells/insert) were seeded into the inserts in EBM/0.5% FBS. Six hours (for migration) or 24 h (for invasion) later, the inserts were washed with PBS, and cells on the top surface of the insert were removed by wiping with a cotton swab. Cells that migrated to the bottom surface of the insert were fixed with methanol and stained by Hoechst 33258 and then subjected to fluorescent microscopic inspection. Cells were counted in 10 random fields at 200× magnification under Nikon Eclipse Ti-U fluorescent microscope.

#### Endothelial cell cord formation assay

Matrigel (250 μl) was added into wells of 24-well plates, and polymerized for 30 min at 37°C. HUVEC were then seeded on the surface of polymerized Matrigel (5.10^4 ^cells/well) and cultured in the presence of NGF (100 ng/ml) or VEGF (10 ng/ml) for 18 h. Tubular networks in each well were photographed using Nikon Eclipse Ti-U inverted microscope before measurement of tubular lengths using NIS element Basic Research (Nikon).

#### Endothelial cell monolayer permeability assay

HUVEC (2.10^5 ^cells/well) were seeded on BD Falcon inserts with a PET membrane/0.4 μm pores (BD Biosciences) in EGM. When cells reached confluence, they were treated with NGF (100 ng/ml) or VEGF (10 ng/ml) in EBM/0.5% FBS for 6 h. The medium was then replaced with EBM/0.5% FBS containing FITC-labeled dextran (70 kDa, 250 μg/ml, Sigma-aldrich). To determine the fluorescence intensity of FITC-Labeled dextran that passed through the insert, 100 μl medium was collected from each well every 15 min during 1 h, and the fluorescence was measured using a fluorescence multi-well plate reader FLx800 (Bio-Tek Instrument) at 483 nm as excitation, and 517 nm as emission, wavelengths.

### Pharmacological inhibition

Inhibition was performed with 10 nM K252a (tyrosine inhibitor of TrkA receptor), 10 μM LY294002 (inhibitor of PI3K), 10 μM PD98059 (inhibitor of MEK 1/2), 10 μM GM6001 (broad spectrum inhibitor of matrix metaloprotease), 5 μM MMP 2 inhibitor I (inhibitor of Matrix Metaloprotease 2) or 0.1 mM L-NAME (inhibitor of nitric oxide synthase). Control cells were treated with DMSO. The concentrations used were based upon the absence of toxicity in HUVEC, as determined by bleu Trypan assay in EBM/0.5% FBS for 24 h. All the inhibitors were from Calbiochem, except L-NAME (Sigma-Aldrich).

### Western blot

Cells were lysed in RIPA buffer (50 mM Tris HCl, 150 mM NaCl, 1 mM EDTA, 1% NP40, 0.25% sodium desoxycholate, 1:100 protease inhibitor cocktail and 1 mM sodium orthovanadate, all chemicals from Sigma-Aldrich) and proteins were separated by SDS-PAGE and then transferred to nitrocellulose membrane (Protran 0.45 μm, Whatman) or polyvinylidene fluoride membrane (Immobulon-P 0.45 μm, Millipore) by liquid transfer.

Blots were blocked in 5% BSA, or 3% non fat skimmed milk, in Tris-Buffer Saline Tween-20 (TBST, 20 mM Tris Base, 150 mM NaCl, 0.1% Tween 20) for 1 h at room temperature, and then followed by incubation overnight at 4°C with the primary antibodies against phospho TrkA (Tyr490), TrkA (Clone 763), phospho NOS (ser1177), NOS, phospho ERK (Thr202/Tyr204), ERK, phospho Akt (Ser473) and Akt. All the antibodies were from Cell Signaling and used at 1:1 000 dilution, except anti TrkA (1:500 dilution, Santa Cruz Biotechnology). After several washes with TBST, membranes were incubated with the horseradish peroxidase-linked anti-rabbit or anti-mouse secondary antibodies (1:10 000 dilution, Jackson Immunoresearch) in 5% BSA in TBST for 1 h at room temperature. Immunoblots were visualized by enhanced chemiluminescence (Supersignal West Pico, Perbio) using chemiluminescence film (Amersham) or Fuji LAS-4000 Mini, according to manufacturers' protocol.

### Nitric oxide (NO) quantification with DAF-2DA

NO quantification was performed as previously described [29]. Briefly, HUVEC were seeded in 96 well-plates (3.10^4 ^cells/well) and cultured for 24 h. Cells were then pretreated in EBM/0.5% FBS, with or without the nitric oxide synthase (NOS) inhibitor L-NAME, for 30 min at 37°C. Cells were then loaded with Diaminofluorescein -2 Diacetate (DAF-2DA )(5 μM final concentration, Sigma-Aldrich) for 20 min. After 2 washes, HUVEC were treated with NGF (100 ng/ml) or VEGF (10 ng/ml) in presence or absence of L-NAME (Sigma) for 2 h. The fluorescence intensity was measured with a multiwell plate reader FLx80 (Bio-tek instrument) using 490 nm as excitation and 520 nm as emission wavelengths. For the fluorescence imagery, cells were seeded on 8 well-Labtek chamber slides (5.10^4 ^cells/well). Following experiment, cells were fixed and mounted and pictures were taken with Nikon Eclipse Ti-U fluorescent microscope.

### Gelatin zymography analysis

The presence and activity of MMP-2 in conditioned medium from HUVEC were analyzed by zymography in 10% SDS-polyacrylamide gel/0.1% gelatin (Sigma-aldrich), according to manufacturer's protocol.

### ELISA detection of secreted VEGF

HUVEC or MDA-MB-231 cells were seeded on 60 mm dishes in complete media. The following day, HUVEC were cultured in 2 ml EBM/0.5% FBS and MDA-MB-231 in 2 ml serum-free MEM in the presence of NGF (100 ng/ml) for 6 h or 24 h. The conditioned media were collected and concentrated with Amicon Ultra-4 10 K (Millipore) according to the manufacturer's instruction. Protein content was then measured with BCA method before ELISA quantification of VEGF according to manufacturer's instructions (Human VEGF Duoset kit from R&D Systems).

### Statistical analysis

The data are presented as the mean ± standard deviation (S.D.) of at least three separate experiments in triplicate. Comparisons between two groups were analyzed using the two-tailed Student's t-test or two-way non-parametric ANOVA test, and significance was established at a p value <0.05.

## Results

### NGF contributes to stimulate breast cancer angiogenesis *in vivo*

**Figure 1:**
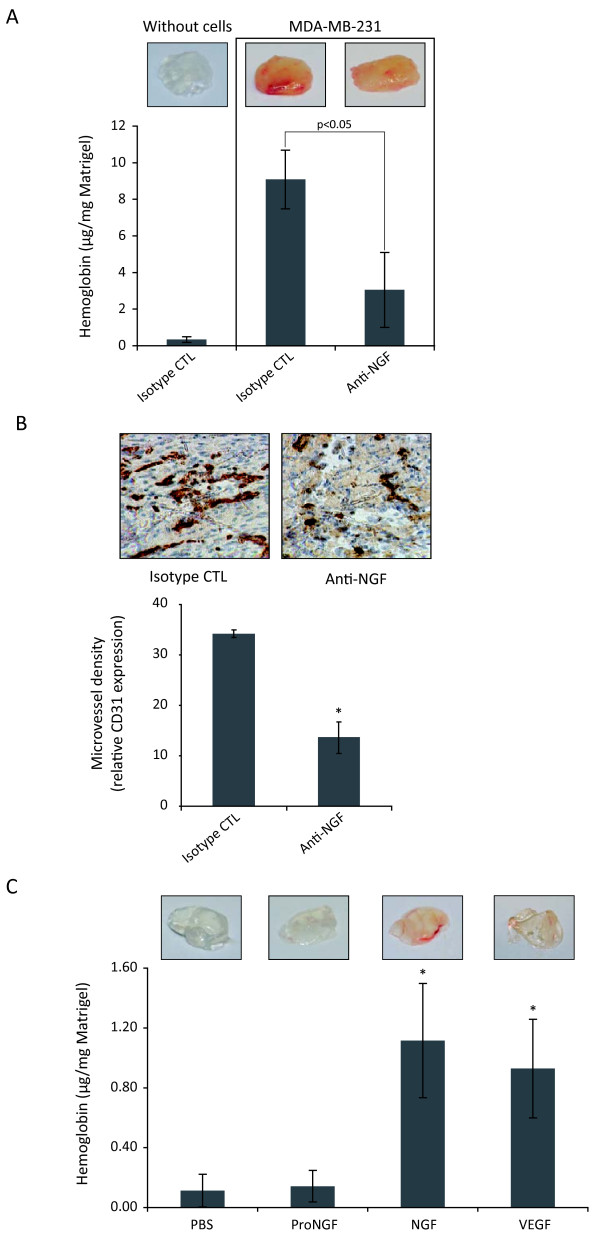
**Angiogenesis assay using Matrigel plugs in SCID mice**. Matrigel containing different reagents was subcutaneously injected into SCID mice as described in materials and methods. (A, B) Matrigel was mixed with MDA-MB-231 breast cancer cells and isotype control or anti-NGF neutralizing antibodies (75 μg/ml); (C) Matrigel was mixed with proNGF (7.5 μg/ml), NGF (3.75 μg/ml), or VEGF (0.375 μg/ml). Angiogenesis was analyzed by quantification of hemoglobin (A, C) and microvessel density (B) as described in materials and methods. Five mice were used for each group and results are the mean of three independent experiments. Student's t-test, *p < 0.01 *versus *control.

To determine the potential effect of NGF in breast cancer angiogenesis, we first performed Matrigel plug assay in SCID mice (Fig. 1A and 1B). Seven days after the experiment, MDA-MB-231 breast cancer cells strongly induced capillary vessel formation in Matrigel plugs, as revealed by hemoglobin content (Fig. 1A) and microvessel density in Matrigel plugs (Fig. 1B). The presence of a neutralizing antibody anti-NGF in the Matrigel plugs decreased about two third the quantity of hemoglobin and microvessel density, suggesting that NGF is strongly involved in breast cancer angiogenesis (Fig. 1A and 1B). Moreover, recombinant NGF induced angiogenesis as efficiently as recombinant VEGF, while proNGF did not induce angiogenesis compared to control (Fig. 1C)

### NGF exerts pleiotropic effects on human umbilical endothelial cells (HUVEC)

**Figure 2:**
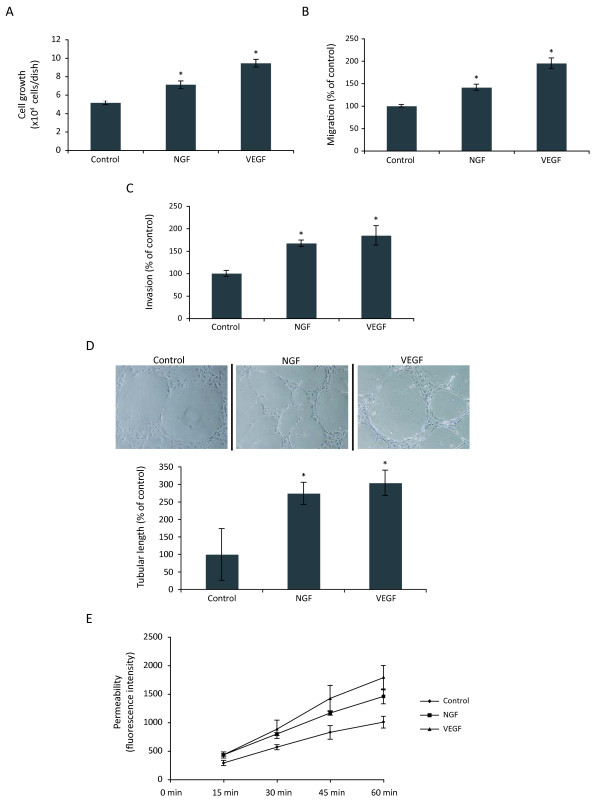
**Pleiotropic effects of NGF on HUVEC**. (A) Growth assay. HUVEC cultured on standard culture plastic were treated with NGF (100 ng/ml), or VEGF (10 ng/ml) for 48 h. (B and C) Migration and invasion assay using Transwells. (D) Cord formation assay on matrigel-coated 24-well dishes. (E) Endothelial cell monolayer permeability assay. The kinetics of 70 kDa Dextran-FITC having passed through the monolayer of endothelial cells was determined by measurement of fluorescence in the lower chambers at different time points. A to D Student's t-test, *p < 0.01 *versus *control. E, ANOVA test, p < 0.05.

The strong involvement of NGF in breast cancer angiogenesis prompted us to determine the effects of NGF on endothelial cells in terms of proliferation, migration, invasion, cord formation and permeability, as all these processes are known to be involved in tumor angiogenesis. We used the well known prototypic angiogenic factor VEGF as positive control. For this, different concentrations of NGF and VEGF were tested; the maximal effects were obtained with 100 ng/ml NGF or 10 ng/ml VEGF, higher concentrations exerted similar effects (data not shown). To simplify the presentation, we show only results obtained with 100 ng/ml NGF or 10 ng/ml VEGF. As shown in Fig. 2A and 2B, NGF stimulated proliferation and migration of HUVEC, but not as strongly as VEGF. It is to be noted that upon 24 h of treatment with NGF, no modification of cell proliferation was observed (data not shown). In contrast, NGF stimulated HUVEC invasion (Fig. 2C) and cord formation as strongly as VEGF (Fig. 2D). Similar to VEGF, NGF increased also the permeability of HUVEC monolayer (Fig. 2E).

### NGF-stimulated invasion of HUVEC involves the activation of TrkA and multiple downstream pathways

**Figure 3:**
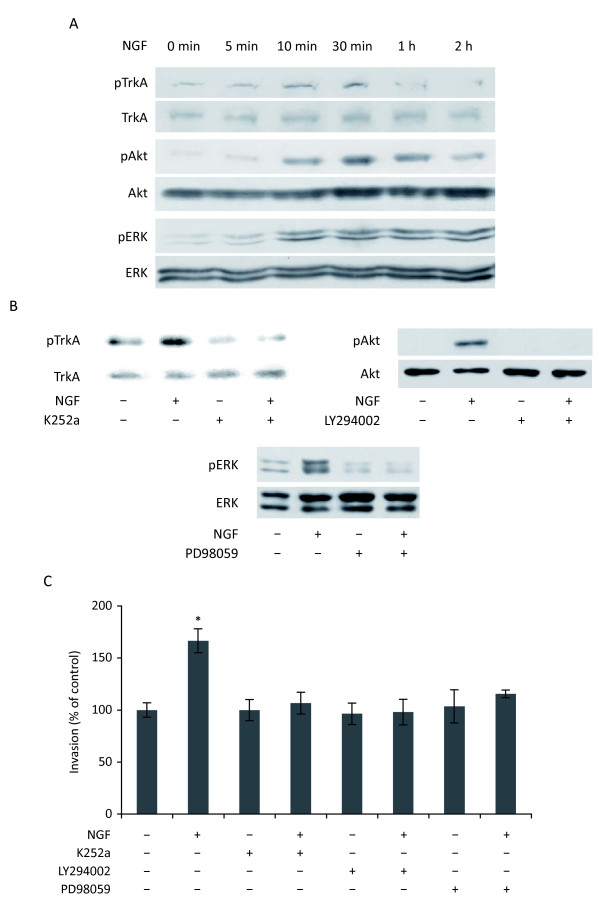
**Involvement of TrkA, PI3K/Akt and MAPK pathways in NGF-stimulated invasion of HUVEC**. (A) Western blot analysis of phospho TrkA (pTrkA), pAkt, and pERK upon NGF (100 ng/ml) stimulation. (B) Inhibition of pTrkA, pAkt and pERK by specific pharmacological inhibitors. HUVEC were pretreated with K252a, LY294002 or PD98059 for 30 min before treatment with NGF for another 20 min. For both A and B, results are representative of at least two independent experiments. (C) Invasion assay using Transwells. HUVEC were pretreated with the indicated pharmacological inhibitors for 30 min and then treated with NGF for 24 h. Student's t-test, *p < 0.01 *versus *control.

As invasion of endothelial cells is an important step in angiogenesis, and as NGF stimulated HUVEC invasion, we decided to determine different signaling pathways involved in NGF-stimulated invasion. As shown in Fig. 3A, upon NGF treatment, TrkA phosphorylation was increased within 10 minutes. Concomitantly, the levels of phospho Akt (pAkt) and phospho ERK (pERK) were increased within 10 minutes and remained high even after 2 h of treatment with NGF. Furthermore, pharmacological inhibition of TrkA (K252a), PI3K (LY294002) and MEK 1/2 (PD98059) (Fig. 3B) totally abolished NGF-stimulated invasion (Fig. 3C). This suggested that NGF-stimulated invasion of HUVEC was mediated by its tyrosine kinase TrkA and the downstream pathways including PI3K and ERK.

**Figure 4:**
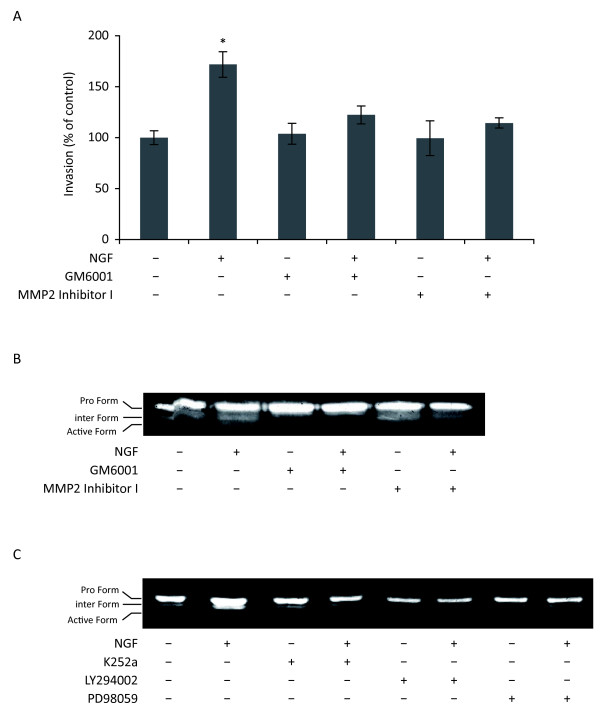
**Involvement of MMPs in NGF-stimulated invasion**. (A) Invasion assay using Transwells. HUVEC were pretreated with the broad spectrum inhibitor of MMPs GM6001 (10 μM) or the specific inhibitor of MMP2 (MMP2 Inhibitor I, 5 μM) for 30 min and then treated with NGF (100 ng/ml) for 24 h. Student's t-test, *p < 0.01 *versus *control. (B and C) Zymography analysis of MMP2. HUVEC were treated with NGF in the presence of different pharmacological inhibitors as described above before zymography analysis. Results are representative of two independent experiments.

Matrix metalloproteases (MMPs) are essential in matrix degradation during cell invasion. We therefore used the MMP broad spectrum inhibitor (GM6001) and the specific inhibitor of MMP2 (MMP2 inhibitor I) to determine the involvement of MMPs in NGF-stimulated invasion of HUVEC. As shown in Fig. 4A, the two inhibitors totally abolished NGF-stimulated invasion. Concomitantly, gelatin zymography analysis (Fig. 4B and 4C) showed that NGF did increase the levels of MMP2 active form in conditioned medium from HUVEC; treatment of HUVEC with GM6001 or MMP2 inhibitor I totally abolished NGF-induced activation of MMP2 (Fig. 4B). In addition, inhibitors of TrkA (K252a), PI3K (LY294002) and MEK 1/2 (PD98059) abolished the NGF-induced active form of MMP2 (Fig. 4C). Together, these findings suggested that NGF-stimulated invasion of HUVEC involved MMPs, particularly MMP2, which was under the control of PI3K and ERK pathways.

**Figure 5:**
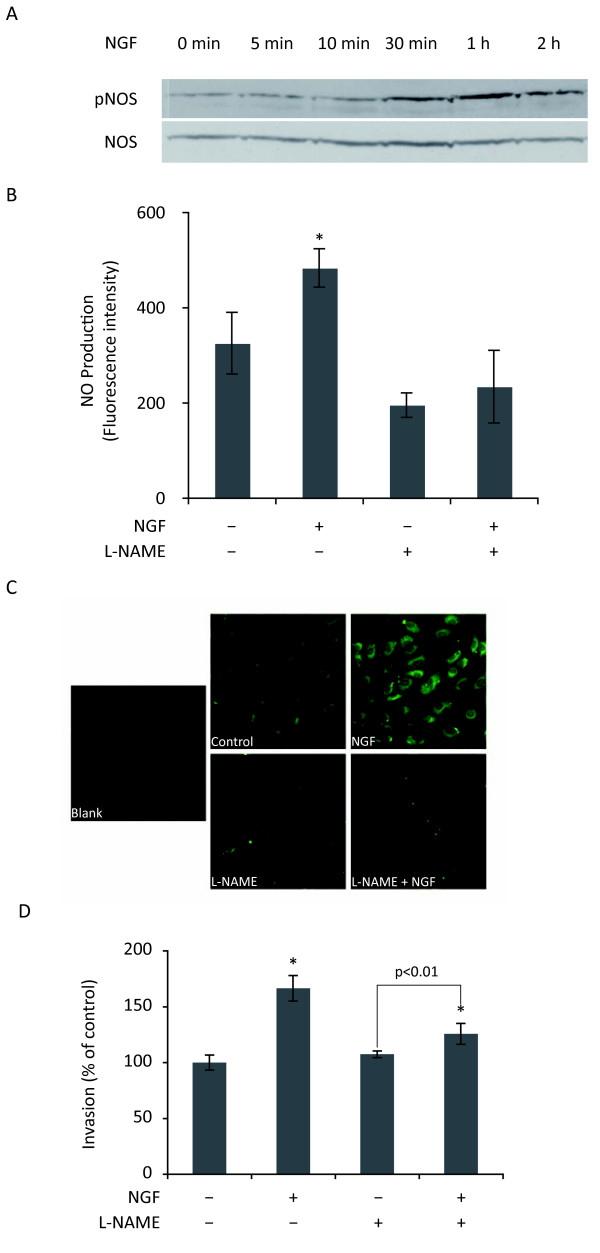
**Involvement of NO synthase in NGF-stimulated invasion**. (A) Western blot analysis of phospho NO Synthase (pNOS) upon NGF (100 ng/ml) treatment. Results were representative of two independent experiments. (B) Quantification of NO levels in HUVEC. Cells were pretreated with NO synthase inhibitor (L-NAME, 0.1 mM) for 30 min, then loaded with DAF-2DA for 20 min before treatment with NGF in the presence or absence of L-NAME for 2 h. (C) Illustration of fluorescence intensity, which represent the levels of NO in HUVEC under different conditions. (D) Invasion assay using Transwells. HUVEC were pretreated with L-NAME and then treated with NGF for 24 h. For both B and D, Student's t-test, *p < 0.01 *versus *control.

PI3K/Akt pathway has been reported to phosphorylate NO synthase (NOS), thus increasing NO production which is responsible for VEGF-induced endothelial cell migration [30,31]. Here, we showed that NGF also increased the levels of both phospho NOS (pNOS) (Fig. 5A) and NO in HUVEC (Fig. 5B and 5C). Moreover, NOS inhibition with L-NAME drastically decreased NGF-induced NO production (Fig. 5B and 5C) as well as NGF-stimulated invasion of HUVEC (Fig. 5D). These data suggested that NGF-stimulated invasion of HUVEC involved the activation of NOS.

### NGF-stimulated breast cancer angiogenesis partially involves VEGF

**Figure 6:**
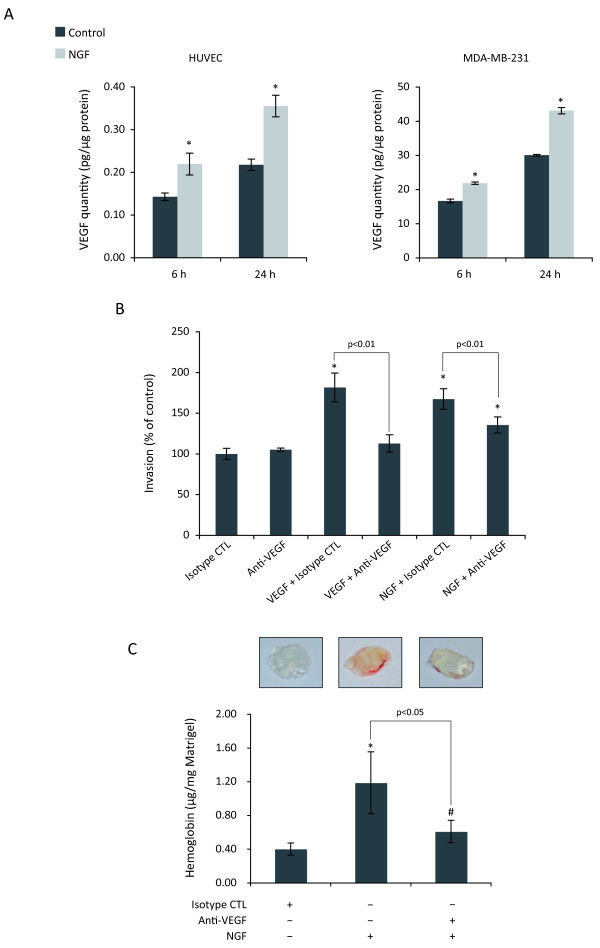
**Involvement of the VEGF in NGF-stimulated angiogenesis**. (A) ELISA quantification of VEGF in conditioned media from HUVEC and MDA-MB-231 cells. Cells were treated with NGF (100 ng/ml) for 6 h or 24 h, conditioned media were concentrated before ELISA assay, as described in materials and methods. (B) Invasion assay using Transwells. HUVEC were treated with NGF (100 ng/ml) or VEGF (10 ng/ml) in the presence of isotype control or anti-VEGF neutralizing antibodies (1 μg/ml) for 24 h. (C) *In vivo *angiogenesis assay. Matrigel containing a mixture of NGF and isotype control or anti-VEGF neutralizing antibodies (37.5 μg/ml) was subcutaneously injected into SCID mice (five mice per group) as described in materials and methods. Hemoglobin in Matrigel plugs was quantified by Drabkin method 7 days after injection. Results are the mean of three independent experiments. Student's t-test, *p < 0.01; ^#^p < 0.05 *versus *control.

It has been described that NGF can stimulate the expression of VEGF in several types of cells including endothelial cells [14], as well as epithelial ovarian cancer cells [32]. We decided to determine the potential implication of VEGF in NGF-stimulated angiogenesis. As revealed by ELISA assay (Fig. 6A), NGF strongly increased the levels of secreted VEGF in both HUVEC and MDA-MB-231 breast cancer cells. Upon 24 h of treatment with NGF, an increase of 63% and 43% of secreted VEGF was observed in HUVEC and MDA-MB-231 cells, respectively. We then determined the involvement of VEGF in NGF-stimulated angiogenesis both *in vitro *and *in vivo *by using an anti-VEGF neutralizing antibody. Although anti-VEGF was able to totally abolish VEGF-induced invasion, neutralization of VEGF led to 50% decrease of NGF-induced invasion of HUVEC (Fig. 6B). Interestingly, similar result was obtained when angiogenesis was determined using Matrigel plugs in SCID mice (Fig. 6C). Collectively, these results demonstrated that NGF-induced angiogenesis was partially mediated by VEGF.

## Discussion

Here, we present *in vivo *and *in vitro *data that give new insights into mechanisms of the involvement of NGF in breast cancer angiogenesis. Using an *in vivo *matrigel model, we showed that strong angiogenesis was set up as early as 7 days after subcutaneous injection of MDA-MB-231 breast cancer cells in SCID mice. Importantly, neutralization of NGF with antibody against NGF reduced more than half of breast cancer cells-induced angiogenesis. These results reinforce our previous findings that treatment of established xenografted mammary tumors with a neutralizing antibody against NGF could reduce the number of endothelial cells in the tumors [24]. Moreover, we found that the *in vivo *angiogenic effect of NGF was similar to that elicited by VEGF; this is consistent with data reported by Cantarella et al. [14] who used chicken embryo chorioallantoic membrane (CAM) as an *in vivo *angiogenesis assay. As VEGF is considered as one of the most efficient proangiogenic factors in breast cancer angiogenesis [6,10], and as NGF is found to be overexpressed in breast cancer [24], our present findings highlight the importance of NGF as a proangiogenic factor in breast cancer.

Tumor angiogenesis involves several processes, including endothelial activation, proliferation, migration and tissue infiltration from preexisting blood vessels that are triggered by specific proangiogenic growth factors produced by tumor cells and the surrounding stroma [33]. These include VEGF [34] and bFGF [35] which have been shown to activate their specific receptor tyrosine kinases, thereby initiating intracellular signaling to drive the angiogenic process. The effects of NGF on endothelial cells have been found to vary according to tissue origin. NGF stimulates proliferation and migration of human umbilical vein endothelial cells, human dermal microvascular endothelial cells and choroidal endothelial cells [14,27,36,37]. In contrast, NGF has no effect on either proliferation or migration of retinal endothelial cells [37]. Here, we showed that NGF strongly enhanced invasion and cord formation of HUVEC with moderate effects on proliferation and migration. Of importance, we showed for the first time that NGF increased the permeability of endothelial cell monolayer *in vitro*. The increased permeability of intratumoral blood vessels is thought to favor tumor cell extravasation during metastasis and to play a crucial role in tumor stroma formation due to leak of plasma fibrinogen [38,39].

As invasion of endothelial cells is one of the important processes during angiogenesis, we decided to determine the signaling pathways involved in NGF-stimulated invasion of HUVEC. We demonstrated that NGF-stimulated invasion was regulated *via *its tyrosine kinase receptor TrkA; this was reinforced by the observation that ProNGF, which acts *via *other receptors (p75^NTR ^and sortilin) than TrkA, had no effect on angiogenesis. Moreover, NGF-stimulated invasion was regulated by TrkA downstream signaling pathways including PI3K and ERK, leading to the activation of MMP2. These findings are partially in agreement with data reported by Park et al [27] in that they observed only the involvement of PI3K, but not ERK, in NGF-induced HUVEC invasion and MMP2 activation. The reason for such a discrepancy is not known, as the same pharmacological inhibitor (PD98059, 10 μM) was used in the two studies; one hypothesis might be the difference of culture medium. Alternatively, as HUVEC are derived from different donors, we cannot exclude some differences due to their origin, despite of the standardized protocol of cell isolation and characterization.

Another interesting finding of our work was the involvement of NO synthase (NOS) in NGF-induced invasion. NOS is responsible for the production of nitric oxide (NO), a highly diffusible signaling molecule, known to mediate a number of functions such as angiogenesis, immune responses and nervous system development [40]. Endothelial NOS (eNOS), is particularly expressed by vascular endothelial cells or surrounding stromal cells and therefore has been a focus of attention in angiogenesis. Thus, using eNOS^-/-^mice, it has been found that NO mediates branching and longitudinal extension of blood vessels in B16 melanomas and that this process is predominantly mediated by eNOS [41]. In cell culture models, eNOS has been described to be involved in migration of endothelial cells [30,31]. eNOS is also involved in the proangiogenic effect of VEGF and prostaglandin E2 [42,43]. VEGF has been reported to stimulate endothelial cell migration by activating Akt which in turn phosphorylates Ser1177 residue of eNOS [30,44]. Here, we found that NGF induced a rapid and persistent increase of phosphorylation of NOS at Ser1177, accompanied by an increase of NO production, suggesting that NGF-induced phosphorylation of eNOS could also involve PI3K/Akt pathway as previously described for VEGF [30,44].

NGF has been described to increase the expression of VEGF in various tissues and cells such as ischemic hindlimb [15,45], nervous system [46,47], epithelial ovarian cancer cells [32] and endothelial cells [47]. Therefore, NGF may exert its proangiogenic effect *via *VEGF. Indeed, we showed NGF can increase the secretion of VEGF in both HUVEC and MDA-MB-231 breast cancer cells. Moreover, NGF-promoted angiogenesis is partially mediated by VEGF, as neutralizing antibody anti-VEGF inhibited about half of NGF-induced HUVEC invasion, as well as angiogenesis, *in vivo*. These data, together with our previous findings of NGF overexpression in breast cancer, suggest that NGF could favour breast cancer angiogenesis in concert with VEGF.

Since anti-angiogenesis strategy using anti-VEGF antibodies such as bevacizumab has been integrated into the treatment of cancers, including breast cancer, the development of bevacizumab-resistant tumors has become more common. Recent studies show that targeting other angiogenesis signaling pathways such as those induced by angiopoietin/Tie-2 may lead to enhanced response in anti-VEGF resistant tumors [48]. In this study, we provided direct evidence that NGF could be an important stimulator for breast cancer angiogenesis. NGF not only stimulates proliferation, migration, invasion and tubule formation of endothelial cells, but also increases the permeability of endothelial cell monolayer. Furthermore, our study allows for the identification of new pathways, such as NO synthase and ERK, in NGF-induced invasion of endothelial cells. Thus, NGF, as well as the activated signaling pathways, should be taken into account for the design of future anti-angiogenic therapeutic approaches against breast cancer.

## Competing interests

The authors declare that they have no competing interests.

## Authors' contributions

RR carried out the cell biology studies, performed the statistical analysis and participated in the draft of the manuscript; EA performed the in vivo studies and participated in the design of the study; CL participated in the cell biology studies and critically read the manuscript; EG participated in the in vivo studies and critically read the manuscript; HH participated in the design of the study and revised the manuscript; XLB conceived of the study, participated in its coordination and drafted the manuscript.

All authors read and approved the final manuscript.
